# Women Tell All: A Comparative Thematic Analysis of Women’s Perspectives on Two Brief Counseling Interventions for Intimate Partner Violence

**DOI:** 10.3390/ijerph19052513

**Published:** 2022-02-22

**Authors:** Danielle R. Shayani, Sara B. Danitz, Stephanie K. Low, Alison B. Hamilton, Katherine M. Iverson

**Affiliations:** 1 Women’s Health Sciences Division of the National Center for PTSD, VA Boston Healthcare System, Boston, MA 02130, USA; sara.danitz@va.gov (S.B.D.); stephanie.low@va.gov (S.K.L.); 2 VA Center for the Study of Healthcare Innovation, Implementation and Policy, VA Greater Los Angeles Healthcare System, Los Angeles, CA 90073, USA; alisonh@ucla.edu; 3 Department of Psychiatry and Biobehavioral Sciences, Los Angeles Greffen School of Medicine, University of California, Los Angeles, CA 90095, USA; 4 Department of Psychiatry, Boston University School of Medicine, Boston, MA 02118, USA

**Keywords:** intimate partner violence, patient preferences, qualitative research, treatment, women veterans

## Abstract

Background: Intimate partner violence (IPV) is a significant public health problem that is commonly experienced by women and associated with psychosocial health issues. Recovering from IPV through Strengths and Empowerment (RISE) is a brief, clinician-administered, variable-length (1–6 sessions), modular, individualized psychosocial counseling intervention developed for women experiencing IPV. We present qualitative feedback and quantitative helpfulness ratings from women patients of the Veterans Health Administration who completed a randomized clinical trial (RCT) comparing RISE to a clinician-administered advocacy-based Enhanced Care as Usual (ECAU; a single structured session consisting of psychoeducation, safety-planning, resources, and referrals). Methods: 58 participants (_M_age = 39.21) completed post-intervention semi-structured qualitative interviews, including helpfulness ratings, at two follow-up assessments (10- and 14-weeks post-enrollment) to assess the acceptability, usefulness, and perceived fit of the interventions for women’s needs. Interviews were transcribed and analyzed using a hybrid deductive-inductive analytic approach. Results: While both the RISE and ECAU interventions were deemed helpful (interventions were rated as ‘highly helpful’ by 77% of RISE and 52% of ECAU participants), differences were identified in perceived impacts of the intervention, application of content, approach to patient-centeredness, and implementation recommendations. Conclusions: Findings shed light on women Veterans’ experiences and preferences for IPV psychosocial counseling interventions. Such knowledge can inform evidence-based, trauma-informed, and individualized care for women Veterans who experience IPV and may have relevance to other populations of women who experience IPV.

## 1. Introduction

Intimate partner violence (IPV), including physical, sexual, and psychological violence, is a complex population health problem. IPV is the most prevalent form of violence against women globally, with recent data from the World Health Organization (WHO) demonstrating that IPV is prevalent across the globe, with more than 640 million women worldwide who experience IPV during their lifetime [1]. According to estimates from the WHO, more than one in four (27%) women have been subjected to physical and/or sexual violence by an intimate partner during their lifetime while one in ten women have experienced physical and/or sexual violence within the past year [1]. The impact of such violence on the physical, mental, and social health of women has been well-documented in the literature [2,3,4]. For example, IPV is associated with numerous emotional and mental health issues including reduced self-efficacy and quality of life, and increased depression, anxiety, posttraumatic stress symptoms, suicidal ideation, and suicide attempts [5,6,7,8,9]. As a result of the multitude of health issues and stress associated with IPV, women who experience IPV often have increased healthcare utilization in both single and multi-country studies [9,10,11,12]. These clinical encounters, regardless of their purpose, provide important opportunities for clinicians to safely and sensitively inquire about IPV and provide therapeutic clinical responses [13,14].

Although routine screening for IPV in the healthcare setting is effective in identifying patients who experience IPV [15], it is critical that screening and response practices include offering referrals to interventions that can appropriately support women following their IPV disclosures. A systematic review by Bair-Merritt and colleagues (2014) reported that promising healthcare-based counseling interventions for women experiencing IPV typically contain elements of advocacy, safety planning, and linkages with community-based resources [16]. Little is known, however, about women’s perceptions of such interventions and the extent to which IPV interventions meet their unique needs and preferences. Some advocacy-based interventions and mental health treatments have been shown to have benefits for women experiencing IPV primarily using quantitative methods [16,17,18]. Quantitative outcomes from randomized clinical trials are certainly critical to evaluating the clinical utility of different interventions, but they are not the only form of evidence. Patient experience and preferences are another important source of evidence [19], but there has been little published about women’s more personal experiences with more structured IPV interventions, including their perceptions of the helpfulness, fit and acceptability of these interventions and recommendations for modifying the interventions or their implementation characteristics based on their personal experiences with the interventions. Investigating qualitative outcomes serves to fill this gap and can inform recommendations for enhancing IPV interventions to better fit and address women’s needs. Although there are many studies examining women’s experiences and preferences for IPV screening and response procedures [20], there is much less research on women’s experiences regarding clinician-administered structured and/or manualized IPV psychosocial counseling interventions.

The need to understand women’s nuanced experiences of these types of IPV interventions comes at an important juncture in the fields of medicine and IPV, intersecting with the integration of patient-centered care [21] and survivor-centered practice [22,23]. These overlapping models of care both emphasize that interventions should be responsive to individual patient/client preferences, goals and values, and individualized needs. Similarly, both patient- and survivor-centered care emphasize personalized positive outcomes– as opposed to focusing primarily on symptom reduction and violence cessation. Thus, in addition to evaluating psychosocial counseling interventions for IPV via traditional examinations of change over time in researcher-selected quantitative measures, it is equally important to also understand the perspectives of the end-users in order to enhance the likelihood of successful uptake of new interventions into routine care [24,25]. It is, therefore, timely to gather women’s perceptions of and feedback on IPV interventions to understand the extent to which structured clinician-administered psychosocial interventions meet their needs as well to inform enhancements in clinical practice and program development to better address IPV.

One group of end-users that is particularly relevant to study are women who have served in the military. Women Veterans are an important population for IPV treatment research as they are at high risk for experiencing IPV, with past-year estimates ranging from 19–37% [26,27,28,29]. Within the United States, women Veterans are more likely to have experienced IPV during their lifetime relative to women who never served in the military [30], and there is evidence that IPV is prevalent among military populations [31,32,33]. Although the field does not yet have a comprehensive understanding of why women Veterans are at heightened risk for IPV, military sexual trauma (i.e., sexual assault and sexual harassment during military services), increases risk for IPV [26]. Moreover, recent IPV is known to contribute to women Veterans’ current health needs above and beyond the impact of other stressors that are prevalent among women Veterans (e.g., military sexual trauma and deployment-related exposures) [34,35,36,37]. Due to the high prevalence of IPV and its negative impacts on health, some Veterans’ healthcare organizations are moving towards integrating care for IPV within existing health services. Within the United States, the Veterans Health Administration is actively implementing screening and intervention for IPV. Understanding women Veterans’ perspectives on IPV interventions is therefore important for informing these efforts. 

The current study begins to fill the aforementioned gaps in the literature by incorporating qualitative methods along with helpfulness ratings to assess women Veterans’ IPV treatment experiences within the context of a larger randomized clinical trial (RCT) study. This RCT targeted women Veterans of the United States Armed Services who were patients of the Veterans Health Administration (VHA) and compared a new empowerment-focused modular-based brief counseling intervention (described in more detail in the Methods section) to an advocacy-based Enhanced Care as Usual (ECAU) intervention [38]. The present study examines post-treatment semi-structured interview data from the parent RCT to understand women Veterans’ perceptions of the IPV interventions with respect to usefulness, acceptability, and perceived impact, and to gather feedback for informing future implementation of IPV counseling programs. Through collecting and reporting on these qualitative interview findings, in tandem with examining participants’ ratings of overall treatment usefulness, there is potential to more thoroughly understand women’s experiences with IPV interventions in a way that is attuned to women’s unique perspectives and preferences. The current study has potential to advance the IPV psychosocial counseling intervention literature by focusing specifically on women Veterans’ experiences with treatment based on their verbal feedback, as opposed to relying on traditional quantitative outcomes alone, as has frequently been the case in prior studies examining new psychosocial counseling interventions for women who experience IPV (e.g., [18,39,40,41]). 

## 2. Materials and Methods

### 2.1. Design and Participants

We conducted semi-structured interviews with participants 10- and 14-weeks following enrollment in a RCT comparing the Recovering from Intimate Partner Violence through Strengths and Empowerment (RISE) brief counseling intervention to an ECAU comparison condition for women Veterans VHA patients who experienced past-year IPV (registered in ClinicalTrials.gov; NCT03261700). The larger RCT and primary quantitative findings have been described elsewhere [38]. In brief, the study took place at an urban VHA medical center in the New England region of the US between October 2018 and November 2020. Data was collected with approval from the VA Boston Healthcare System’s Institutional Review Board (IRB #3078).

In total, 60 women VHA patients participated in the larger RCT. Participants were recruited via flyers posted in the hospital, self-referrals, clinician referrals, and recruitment letters mailed to VHA patients. Flyers defined IPV and described the treatment study. Recruitment letters broadly described a Women’s Treatment Preferences study taking place at the local VHA medical center. Interested women were screened by phone for eligibility criteria, in which a trained research assistant (DS) administered a five-item IPV screener that has been validated with women Veterans [26] and asked basic demographics and mental health questions. Women were included if they reported: (1) past-year IPV (i.e., one or more instances of physical, sexual, and/or psychological IPV on the Revised Conflict Tactics Scale (CTS-2) [42], (2) past-year VHA care, (3) being at least 18 years of age, and (4) willingness to have sessions audio recorded. Women were excluded if they endorsed active past-month symptoms of mania or psychosis and/or homicidal or suicidal ideation warranting hospitalization. 

### 2.2. Procedures

**Figure 1:**
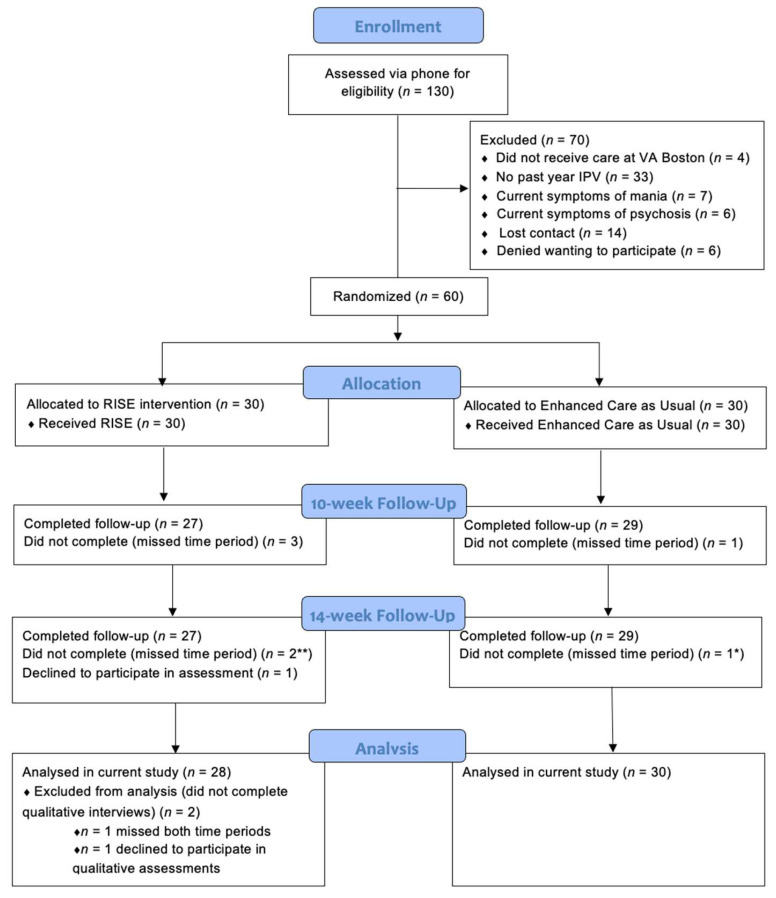
Participant flow through the study, including qualitative interview for analysis. Note. * In the Enhanced Care as Usual (ECAU) condition, the participant who missed the 10-week assessment was not the same participant who missed the 14-week assessment. Thus all 30 ECAU participants had interview data analyzed in the current study. ** Of the RISE participants, one of the three participants with missing data missed only one of the two assessments. Thus 28 RISE participants had interview data analyzed in the current study.

The recruitment, intervention, and analysis team included trained individuals from a variety of educational backgrounds. More details are provided throughout the manuscript. For the RCT, trained bachelor-level research assistants and a doctoral-level clinical psychologist conducted screening, consent, and assessment procedures. Women who were eligible following a phone screening scheduled an in-person enrollment session, and those who were ineligible were provided with appropriate alternative resources. At the enrollment session, women provided written informed consent and filled out a packet of questionnaires for their pre-treatment assessment. They were randomized to receive RISE or ECAU, which they received immediately following their pre-treatment assessment (for additional details on primary and secondary quantitative measures and findings, see Iverson et al.) [38]. Most participants (*n* = 50) received sessions and assessments in person at a local VHA medical center as was intended with the original study design, and 10 participants received one or more sessions and/or assessments via telehealth due to the COVID-19 pandemic. There were no significant differences in pretreatment sociodemographic characteristics for the ten participants who had telehealth sessions versus those who did not as part of the larger study (all *p*-values > 0.05). The study’s randomization procedures remained the same following the onset of COVID-19 and resulted in an even distribution of participants to each of the two intervention conditions. Participants completed follow-up assessments 10- and 14-weeks following enrollment, which included semi-structured qualitative interviews at each time point. Participants were compensated $25, $50, and $75, respectively, at each of the three assessment timepoints. The compensation was commensurate with the amount of time required to complete the assessments and was approved by the Institutional Review Board. Participant flow throughout the study is shown in Figure 1. The current study focuses on helpfulness ratings and qualitative findings from the post-intervention semi-structured interviews conducted at both the 10- and 14-week follow-up assessments.

### 2.3. Intervention: Recovering from IPV through Strengths and Empowerment (RISE)

RISE is a variable-length, modular-based, survivor-centered and trauma-informed intervention that ranges from 1–6 sessions and was administered over the course of 10 weeks in the current study. RISE is rooted in principles of Motivational Interviewing [43] and empowerment, and consists of six modules, namely: (A) Safety Planning, (B) Education on Health Effects of IPV and Warning Signs, (C) Improving Coping and Self-Care, (D) Enhancing Social Support, (E) Making Difficult Decisions, and (F) Connecting with Resources and Moving Forward (see Iverson, Danitz et al., 2021 [44] for more information regarding the RISE modules). During the initial session, RISE clinicians provided an overview of RISE and its structure, introduced the concept of self-efficacy, and invited the woman to share her experiences with IPV and her goals for treatment. Next, the participant selected a module to focus on during the remainder of the session and set a behaviorally-specific goal related to the module to focus on following the session. Subsequent sessions focused on a safety check-in, self-efficacy tracking, goal review, and additional module selection and goal setting. Each module includes specific handouts, exercises, and related goal setting. Modules do not need to be conducted sequentially, nor do all modules need to be completed, and modules can be repeated, as determined by the woman receiving RISE. Participants chose how many RISE sessions to receive, up to the six-session limit. Participants were connected to other VHA and community services, as desired. Additional information regarding the RISE philosophy, content, and structure are reported elsewhere [44]. 

### 2.4. Intervention: Enhanced Care as Usual Condition (ECAU)

ECAU is an advocacy-based intervention that incorporates best-practice recommendations for addressing IPV in VHA. Participants in the ECAU condition received a one-time, 60-min intervention with a provider. At the start of the session, participants were given VHA’s IPV educational brochure to provide psychoeducation about different forms of IPV, prevalence rates of IPV, and the effects of IPV on physical, mental, and social health. ECAU provides information about safety planning, including an optional brief safety plan that can be completed with the provider. Participants in the ECAU condition receive information about local and national resources, including both VHA and community resources, and have the opportunity to connect with these resources and receive referrals as relevant and requested by the woman. 

### 2.5. Interventionists 

Interventionists were VHA clinical providers, including one social worker and three clinical psychologists, with relevant clinical experience and expertise in trauma-informed care; all were women. Providers delivered both interventions within a primary-care setting and an outpatient mental health clinic. Providers participated in weekly consultation with the RISE intervention developer, and both RISE and ECAU sessions were reviewed by the study principal investigator (KI) and discussed to ensure fidelity to the respective treatments. 

### 2.6. Approach

Semi-structured audio recorded interviews were conducted at the 10- and 14-week post-treatment assessments by qualitatively-trained study staff. The interviews were conducted at both timepoints, as it was possible that women’s perspectives or recommendations could be different at the 14-week assessment because they have had more time to try out what they learned in the interventions. Author AH, a medical anthropologist and implementation scientist, provided oversight and overarching guidance for data collection and analysis. An interview guide was developed based on the treatment development and implementation literature, and Veteran and clinician stakeholder input. Interview questions were semi-structured in nature, with a focus on elucidating participant experiences and attitudes about their respective intervention. Of note, the interview guide had been piloted and refined during a pilot study [44]. All interviews were conducted by the project manager (SD), a doctoral level female psychologist with training in qualitative interviewing, or the research assistant (DS), a bachelor’s level female with training in qualitative interviewing. The interview guide included questions querying their overall experiences with acceptability of the intervention (e.g., “What did you like about the intervention?”, “What didn’t you like about the intervention?”), perceived impacts of the intervention (e.g., “How, if at all, do you think participating in the intervention has impacted you?”), application of content (e.g., “how, if at all, do you use the information from the intervention in your everyday life?”), modifications needed to enhance acceptability and helpfulness of the intervention (e.g., “What do you wish you could have changed about the intervention to make it more useful to you?”), and additional implementation recommendations (e.g., “Is there anything you’d like us to change about how the intervention is delivered?”). We added items about experiences with treatment during the COVID-19 pandemic (e.g., “To what extent, if at all, has the current COVID-19 pandemic impacted your ability to participate in this intervention?”). In addition to these open-ended questions, the interviewer asked women to rate “how helpful [they] found the intervention” on a 5-point Likert scale (1 = highly helpful, 2 = somewhat helpful, 3 = neither helpful nor unhelpful, 4 = somewhat unhelpful, and 5 = highly unhelpful). Interviews (mean = 18 min) were transcribed verbatim. 

### 2.7. Data Analysis Strategy

Interviews were analyzed using a hybrid deductive-inductive analytic approach, following an established multi-step approach for efficient and effective identification of key findings from the interviews. The analysis team was trained and led by AH and consisted of two doctoral-level clinical psychologists and a bachelor’s level research assistant (KI, SD, and DS). The multi-step approach followed common rapid analytic methods in intervention development and implementation research [45,46]. First, a template was developed deductively with topics from the interview guide questions (i.e., 1–2 topics per primary interview question). Each analyst independently summarized the same three transcripts, to test out the template and to establish consistency of summaries across analysts. Summaries were compared to one another, and refinements were made to the template and the summarizing process. Then, all transcripts were summarized using the template, generating one summary per transcript that contained key content points and exemplary quotations (as well as other observations when relevant, i.e., any content that fell outside of the deductively derived topics) from the transcript. Interview transcripts and summaries were reviewed by the qualitative lead, who provided regular feedback regarding completeness and consistency. 

Next, the team compiled the summaries into matrices, one matrix each for the RISE and ECAU participants, and used matrix analysis [47] to display content and explore themes inductively within and across conditions at the 10- and 14-week assessments. Themes for the 10- and 14-week assessments were found to be consistently similar within both the RISE and ECAU conditions and findings for the two timepoints were subsequently merged. Findings were then summarized for each condition, highlighting similarities and differences in perceptions across the two conditions. Research assigned participant numbers accompany exemplary quotes to protect participant confidentiality. 

## 3. Results

Of the 60 participants from the RCT, 58 (28 = RISE, 30 = ECAU) participants completed the interviews at the 10- and/or 14-week follow-up assessments and are included in the analysis (RISE: *n* = 1 did not complete either the 10- or 14-week assessment and *n* = 1 did not complete the interview portion at either assessment point, resulting in *n* = 28; ECAU: *n* = 2 did not complete 10- or 14-week assessment but because these were different participants, there was interview data at one of these timepoints for all 30 ECAU participants). Figure 1 provides a summary of participant flow throughout the study. Table 1 describes the baseline sociodemographic characteristics and IPV characteristics of the current sample by condition. A higher percentage of participants in the RISE condition were employed full-time. There were no other group differences in sociodemographic or IPV characteristics (all *p*-values > 0.05). Three interviews were not transcribed due to recording not being feasible and two interviews from each condition were accidently deleted prior to being transferred and transcribed. In these instances, we relied on memos and written notes for these participants. 

In the ECAU condition, all participants (*n* = 30) received a one-session intervention. In the RISE condition (*n* = 28), 23 women (82.1%) completed at least two sessions, 21 (75%) completed at least three sessions, 17 (60.7%) completed at least four sessions, 14 (50%) completed at least five sessions, and nine (32%) completed all six sessions. The percentage of RISE participants selecting each module were as follows: Improving Coping and Self-Care (75%), Education on Health Effects of IPV and Warning Signs (57.1%), Enhancing Social Support (57.1%), Connecting with Resources and Moving Forward (57.1%), Making Difficult Decisions (50%), and Safety Planning (35.7%).

In terms of quantitative ratings of overall helpfulness (on a Likert scale of 1–5, with 1 = highly helpful, 2 = somewhat helpful, 3 = neither helpful nor unhelpful, 4 = somewhat unhelpful, 5 = highly unhelpful), none of the participants reported less than adequate levels of perceived helpfulness (i.e., no ratings of 3 or higher). The majority of RISE participants (76.9%) and just over half of ECAU participants (51.7%) rated the intervention as ‘highly helpful.’ The remaining participants in both groups rated the intervention as ‘somewhat helpful’ (23.1% for RISE and 48.3% for ECAU). 

Similarly, qualitative results indicated that all participants, regardless of their condition, were appreciative of having an intervention available to them; the majority were not aware of other alternative counseling interventions for IPV. Both interventions were perceived as generally acceptable, although several differences were identified in terms of perceived impact, application of content, patient-centeredness, and recommendations for implementation.

### 3.1. Perceived Impact of the Intervention

Women in both conditions reported that they found the intervention to be helpful, but the specific ways in which the interventions were perceived as helpful tended to differ between the two conditions. RISE participants shared more positive impacts on emotional health and psychosocial well-being, whereas the ECAU participants tended to highlight more practical impacts. Specifically, women in the RISE group reported the intervention impacted the way they felt about themselves, including feeling more empowered, confident, and independent than when they started the intervention. For example, women in RISE shared that they felt more confident to think about their relationships and their personal needs differently and take a more active role in improving various areas of their life, including their relationships, and prioritizing their physical and mental health. For instance, a RISE participant described,

“*RISE has empowered me to take much more control over the interpersonal relationships in my life (RISE, 101)*”.

While discussing making the decision to leave her relationship during the study, another participant shared,

“*It’s definitely made me realize and get some self-worth back and feel more empowered by my decisions (RISE, 102)*”.

Similarly, another woman in RISE shared that,

“*I didn’t realize I wasn’t taking care of myself or advocating for myself much. RISE gave me that confidence to start doing that (RISE, 103)*”.

Confidence and independence were increased through independent thinking and decision-making. As noted by a RISE participant when discussing her desire to be more financially independent,

“*In the past I wanted to be separated from him, but I realized I couldn’t do that. Now I am working towards that financial independence (RISE, 104)*”.

These types of benefits were not typically noted among women in the ECAU group. 

In terms of potential mechanisms of these changes, women in the RISE group, many of whom opted to participate in multiple RISE sessions (mean sessions received was 3.8 sessions), shared that reflecting over time with their RISE provider and engaging in exercises within specific modules, setting goals related to the module, and practicing between sessions helped them acknowledge their strengths and restore self-esteem,

“[RISE] *opened my eyes and after every session, I would think about what the session was about. When I’m driving or when I have a quiet period, I would realize, ‘oh yeah, I do have this quality’ or ‘oh yes, I’ve been able to handle this problem.’ So, it made me see that I wasn’t as awful as I thought I was or as inadequate as I thought I was. It made me realize I’ve accomplished a lot more than I had realized (RISE, 104)”*.

Women in the ECAU group described the intervention as having helpful impacts too, particularly in terms of increasing their overall knowledge about IPV and the variety of resources they could potentially make use of now or in the future. In terms of knowledge, multiple women discussed the value of learning about and paying homage to the different forms of IPV and their impacts on health, which helped them to validate their IPV experiences. One woman stated,

“[the intervention] *covered all bases including every aspect of abuse in a relationship: physical, sexual, and emotional. I feel as though emotional abuse can be more detrimental than physical sometimes (ECAU, 105)*”.

Another woman noted,

“*It impacted me by teaching me that I’m not responsible and I don’t deserve specific treatment, like abuse. Whether it’s physical, emotional, mental (ECAU, 106)*”.

This information was typically perceived as normalizing and helped women understand they were not alone in their experiences. Notably, the ECAU participants consistently commented on the impact of the breadth of information provided within the brochure that was used to help facilitate the information sharing and elicit discussion during the session,

“*The wealth of information and contact information for resources in the pamphlet I took it home, and I poured over it for days. I googled every single thing, whether I thought I needed it or not. Stuff like that. But that whole thing made me feel not alone in this (ECAU, 107)*”.

The intervention and brochure in particular led this participant to seek out additional information and resources. 

### 3.2. Application of Content

Participants in both conditions reported that they applied tools and resources provided through the interventions. However, women in RISE endorsed more active changes in their daily lives through incorporating self-care, activating social support, and being more assertive in relationships, whereas women in ECAU applied tools specifically from the informational session and brochure, such as accessing IPV-relevant services and increasing awareness of IPV-related health impacts. 

RISE participants identified several topics and handouts that they directly applied and utilized in their daily lives. Some notable applications were self-care, using decisional balance exercises from the Making Difficult Decisions module and applying strategies from the Enhancing Social Support and Safety Planning modules. For example, when asked about intervention applications, one participant shared,

“*I still have and use the handouts, especially the self-care handout. Sometimes it’s hard for me to do self-care, but with handouts it reminds me that I don’t have to do anything major. It can be something simple, like aromatherapy. Even if I don’t have the funds to go out. Just little things. It definitely adds up (RISE, 103)*”.

Another participant described utilizing an exercise that is part of the Making Difficult Decisions module after participating in RISE:

“*I have been weighing my pros and cons sometimes before I make difficult decisions (RISE, 108)*”.

Increased skills around making decisions led to more confidence in her ability to make good decisions for herself.

Moreover, other RISE participants elaborated on the importance of social support as a mechanism of coping and maintaining safety. During and following the receipt of RISE, women engaged with social support, noting:

“[RISE] *led me to reach out to a couple people just to make sure we were covered in case I had to use my safety plan. For me, that was a little bit more of going outside of my norm to ask for help, especially for the relationship situation (RISE, 109)*”.

Likewise, another woman described utilizing content and concepts from the Education on Health Effects of IPV and Warning Signs module to improve her physical and mental health, noting:

“[RISE] *really impacted my physical health so much. I try to make plans to workout more or exercise or do things that were more towards my physical wellness. Emotionally, RISE kind of helped me to stabilize my emotional roller coaster on some days (RISE, 110)*”.

These examples of applying RISE concepts, resources, and skills emphasize some of the RISE intervention’s unique mechanisms of helpfulness, appropriateness, and usability.

Participants in ECAU also provided examples of applying what they had learned from the intervention, although the applications were somewhat less concrete and mostly involved the information and resources discussed in the session and the associated brochure. An ECAU participant noted,

“*I liked that she gave me the pamphlet and went through it with me during the session. It was helpful. She gave me resources to help me and places to go in the VA for certain things (ECAU, 111)*”.

She elaborated that her provider helped connect her with case management, and she had since been utilizing their services which she found valuable in moving towards practical goals. 

The sentiment that the pamphlet was a helpful tool resounded amongst many participants in ECAU. Another ECAU participant echoed that,

“*just to have that information was comforting (ECAU, 112)*”.

This participant emphasized that the statistics about other women experiencing IPV, as well as the normalization of common reactions and health consequences of IPV, also played a role in providing social connectedness, as it made her feel comforted and not alone. Participants in both interventions often disclosed that they did not fully comprehend the seriousness of the abuse in their relationship or that they were not to blame for the abuse. Similarly, another ECAU participant poignantly appreciated the brochure and gave credit to the intervention for impacting the decision to leave her partner, stating:

“*I felt like I had resources within the brochure. You gave me a lot of resources to use to feel better and get help to leave. So that was good (ECAU, 113)*”.

Participants in ECAU utilized the pamphlet to better understand their situation:

“*I’ve been evaluating my situation more clearly with information from the brochure (ECAU, 114)*”.

This participant reflected that the validating nature of their provider helped normalize her IPV experiences and therefore enabled her to be more open to recognizing the corresponding health impacts.

### 3.3. Patient-Centered Approach

Participants in both the RISE and ECAU conditions expressed appreciation for the patient-centered nature of the interventions. Further, RISE participants noted that they appreciated the flexible yet structured and contained nature of RISE,

“*I liked that we had a menu. Every week I got to pick because then I don’t have to sit there and wonder, ‘okay, what do I want to talk about today.’ She gave me options. If I didn’t want to deal with the feelings today and instead deal with coping with stress, that option and choice was very good for me (RISE, 108)*”.

Similarly, another participant described how selecting the focus of her session was empowering because it gave her agency in her own treatment, noting:

“*I got to pick the topic based on what I was going through. That was very good. It helped me to think, ‘okay do I need to focus on this area a little bit more or that area.’ So, I liked that I was choosing myself but talking to somebody with expertise at the same time (RISE, 110)*”.

Another RISE participant echoed this sentiment, stating:

“*I liked the menu items in the beginning that we covered. It gave me a chance to figure out where I was currently at in my mental state, and where I am at in that relationship. So, I really liked that. It definitely catered to what I needed instead of, this is what we are going to do today regardless of what I feel. So, I liked that (RISE, 115)*”.

In both groups, participants liked that the interventions were tailored to them as women Veterans who experienced IPV. In particular, they valued that the interventions had general resources as well as specific resources related to their identities as women and as Veterans. An ECAU participant further describes this in reference to a referral that she was provided to a Post-9/11 social worker:

“*It is tailored to us, which is more specific, it’s more beneficial than just some random* [other resource]*... oh there’s an actual office just for Veterans like me, and you should go there (ECAU, 116)*”.

The participant reflects how targeting women Veterans specifically and individualizing the intervention to assist in their explicit needs contributed to her high level of satisfaction with the intervention. This may encourage women to feel more comfortable utilizing follow-up services as they reflect on their individual needs. Another ECAU participant emphasized the value of utilizing a women’s space within the VHA. She stated,

“*I wasn’t aware there was a space for us women with partner issues. I was very happy that I was able to get that information because I want to say that I haven’t received that anywhere. Then, after the session, I felt like I had a space to do so (ECAU, 117)*”.

### 3.4. Implementation Recommendations

Participants were asked to describe any changes they would make to how the intervention was delivered that would have improved their overall experience with the intervention or that might help other women experiencing IPV. Across both conditions, there was a desire for more sessions; however, this desire was more common and pronounced for ECAU participants, and the rationale for wanting more sessions differed between groups. The RISE participants who wanted more sessions indicated a desire to continue to further their progress, as they had experienced initial positive impacts from the intervention, and they wanted to continue their growth. For example, one woman in the RISE group shared,

“*I wish that it could have been more thorough and longer. Like if it became a permanent thing, I am sure that would fix the problem, but just the one a week and because it did have such a big impact on me, there wasn’t enough of it because now here I am, and now I’m on the other side of that, and there’s still a lot of work that needs to be done (RISE, 102)*”.

This suggests that RISE might be more impactful if participants had the option to engage in more sessions. Later in the interview, she described in detail why it would have been helpful for her to have more sessions,

“*I wanted to do all the topics and there were six, but there were only six sessions. I wish it could have been longer. Some weeks I really wanted to go back over one topic, but then I wouldn’t be able to, I’d have to cut one out (RISE, 102)*”.

Another RISE participant indicated that she wanted several more sessions

“*because that really helps you set goals and then it helps you keep them. To go back and look at the first goal that you set, you know, 20 weeks prior, would be extremely helpful to kind of just remind yourself like you made this progress, don’t let go of it. Just to kind of build habits so it is not like something that you do once, and you move on and you don’t do it again (RISE, 118)*”.

This suggests that including more RISE sessions might further instill the skills and applications implemented in RISE. 

The ECAU participants wanted more sessions because they wanted the opportunity to further reflect on their IPV experiences and gain more in-depth benefits from the intervention. ECAU focused primarily on providing information and resources, validation, and safety planning. Although the intervention was generally deemed acceptable, many ECAU participants expressed that they wished they had a designated time and space to reflect on their IPV experiences and its impact on their lives in more detail, and wished they had more sessions to reflect on things that arose or were triggered during their ECAU session. One participant stated that she would have benefitted from “*more time to process things (ECAU, 112)*”. 

This participant went on to mention that having more sessions could have strengthened the impact of the supportive messaging that is part of the intervention:

“*I feel like if I had more sessions, the more stuff would stick. Just being reassured that things weren’t my fault or things like that would stick more (ECAU, 112)*”.

Although ECAU participants appreciated and utilized the informational and resources-based intervention, a number of participants reported feeling emotionally triggered or overwhelmed by the extensive information provided in a single session:

“*I think that the brochure she showed was really good, but with the brochure, I noticed after being here, I had a really hard day after that, and the day after. I think it was because of the brochure and how simplified it was (ECAU, 119)*”.

This participant recommended that some women may appreciate an offer for a scheduled phone or in-person check-in following the session to validate, problem solve, and answer questions. Another ECAU participant echoed,

“*Just, it just seemed a little overwhelming. I actually feel like, hmm, maybe to have a couple more sessions so you don’t feel so stressed (ECAU, 120)*”.

This participant recommended breaking up the ECAU intervention into multiple sessions to give participants the opportunity to consume the IPV information at a more manageable pace, hence reducing their stress and enhancing their ability to benefit more from the intervention. Another ECAU participant voiced that having multiple sessions would have helped her feel more comfortable and open with her clinician. She stated,

“*I definitely would have said maybe a couple more sessions to get it all out to that one person and see that one person a few times and start to kind of build a relationship (ECAU, 121)*”.

This participant suggests that having more sessions might have facilitated a stronger therapeutic relationship which might have led to higher acceptability of the treatment. 

An additional domain related to implementation recommendations was the desire for telehealth options. Prior to the COVID-19 pandemic, both interventions were delivered in person. Due to the COVID-19 pandemic, RISE and ECAU were converted to telehealth for the final 10 participants in the study. Before the COVID-19 pandemic, women in both interventions consistently expressed interest in utilizing telehealth as an option to limit barriers to care that are common among women with recent IPV experiences. As detailed here by a participant in the RISE group,

“*Maybe doing telecommunication or skype videos. Cause I think that would have a greater impact...you could reach a lot more people. If they can’t get to you, they can literally just use their phone or computer and still talk to that person as long as they are in a safe space. And maybe they don’t feel comfortable* [in the medical center] *and they feel comfortable in their house talking (RISE, 122)*”.

Once the interventions were adapted to telehealth, interview questions were modified to inquire about their experience of receiving an IPV intervention during the pandemic. One woman who transitioned from in-person RISE sessions to telehealth suggested that telehealth was a better option for her as she could more easily attend sessions (in this case, avoid transportation barriers with getting to the medical center),

“*I almost think it’s better over the phone because I am in my own comfortable space. I am not rushing to get into a hospital. Getting through traffic. I was resistant to doing therapy over the phone, but now, I have adapted to it, and I actually prefer it more (RISE, 123)*”.

Another participant in the RISE condition shared that she experienced an unanticipated problem with telehealth related to privacy:

“*I was completely comfortable with telehealth. The only time I wasn’t was when I was at my boyfriend’s mom’s house. I was like in my car doing it. It was my own fault for forgetting that I had the session, but my window was down, I think someone might have overheard me. But normally if I am home, I am completely comfortable (RISE, 118)*”.

Although some participants were hesitant to participate in teletherapy originally, as some had difficulty locating a private space, most women ultimately found it more palatable and feasible since it cut down time constraints and other barriers to care. 

## 4. Discussion

Women who have experienced IPV and seek IPV treatment have a variety of unique needs and preferences [24,48,49]. Through qualitative exit-interviews conducted in the context of a larger RCT of a new brief counseling intervention, the women Veterans in this study provided insightful feedback that can inform IPV programs and clinical practice with women Veterans who experience IPV and may have relevance to other populations who experience IPV. Both interventions were deemed as generally acceptable, as demonstrated by both quantitative helpfulness ratings and women’s subjective experiences and feedback regarding the counseling interventions received. Findings across conditions highlighted women’s preferences for, and benefits of, receiving tangible resources, a sense of social-connectedness, patient-centeredness, flexibility, and a sense of empowerment surrounding their treatment plan and goals. 

Our findings add to prior qualitative work investigating IPV survivors’ experiences with disclosing and discussing IPV in general mental health contexts [11,50] by adding the perspective of women Veterans with past-year IPV experiences who participated in structured psychosocial counseling interventions during an RCT. Sorrentino and colleagues [11] gathered qualitative feedback regarding IPV screening, disclosure, and response within the VHA healthcare setting from women Veterans who had lived experience with IPV and received past or ongoing mental health care (not necessarily for IPV) [11]. Results from the current study demonstrate that women’s experience of RISE replicates and extends several of Sorrentino’s [11] findings, including the importance of patient-centered IPV treatment that includes respect for clients’ self-determination and flexibility (i.e., content focus and number of sessions included in the intervention) and promoting safety and access to outside resources. In the present study, women particularly appreciated RISE’s flexibility, patient centeredness, and tailored resource provision (i.e., discussing in detail those resources that most map on to the concerns and stated needs of women). Current study findings suggest that clinicians addressing IPV would benefit from utilizing these principles in the context of psychosocial counseling for IPV and in the context of identifying IPV in the context of mental health care. Overall, participants who received RISE appreciated being able to choose the total number of sessions attended (between 1 and 6) and each session’s focus (i.e., personalized module selection). This finding aligns with the growing evidence base supporting patient preferences for flexible and modular mental health treatment models [51,52].

Interviews in the current study revealed that the additional flexibility during the COVID-19 pandemic (i.e., receiving the intervention via telehealth) across both interventions was important, feasible, acceptable, and safe. Trauma-informed telehealth strategies (use of headphones for participants; environmental safety checks that included yes/no questions to assess for presence of another adult or child over 2 years of age; participant use of code words to change the subject if a partner or other person enters the room or is in earshot) were applied to bolster women’s privacy, confidentiality, safety, and comfort addressing IPV via telehealth [53,54]. We also brainstormed with women, when relevant, locating private and safe locations for participating in IPV intervention sessions. Women sometimes took calls or videos from their (parked) cars which we endearingly referred to ‘counseling in cars’ [53,54]. This demonstrates support for delivering RISE and other IPV interventions face-to-face and via telehealth, which is important not only in the context of a pandemic or other public health crisis and is often compatible with patients’ busy and often hectic lives [55]. It also provides another way to explicitly offer choice and voice in IPV care.

Women across both intervention conditions shared during the interviews that they utilized a number of additional resources and services as a result of direct linkages from the intervention. IPV screening and response literature confirms the importance of tangible IPV resources [56,57,58,59], and our study extends this by highlighting concrete resources and applications that are important in formal IPV counseling. Women Veterans in the current study across both conditions noted that it was helpful that providers had a comprehensive list of relevant services at hand as well as help clients personally connect with outside resources in the community (i.e., making a call together), which may have contributed to client follow through. Additionally, echoing findings from Dyer and colleagues (2020), women in this study across both intervention conditions valued the gender tailoring and the importance and attention placed on women Veterans within the treatment (e.g., resources specific to women, and to Veterans) and suggest that this type of tailoring is important in IPV counseling [60].

A number of the participants across the groups spoke about the importance of the IPV interventions for enhancing a sense of social-connectedness and social support. The literature reveals that social support is instrumental in fostering quality-of-life and healing, including in the context of abuse and IPV [61,62,63]. Women in both intervention groups noted that they felt comforted and supported through learning about the wide prevalence of IPV (e.g., by not feeling alone in their experiences), while women in RISE shared that they reached out to their support systems and the people they listed on their safety plan to feel connected. It is possible that RISE’s “Enhancing Social Support” module and accompanying worksheets encouraged women to reach out to support systems including friends and family members in a more concrete way than women in the ECAU condition, perhaps contributing to positive quantitative psychosocial outcomes and more overall satisfaction with the RISE intervention observed in the parent study [38]. 

Participants in both intervention conditions endorsed a preference for being offered multiple sessions and the perceived benefits of reflecting weekly and engaging in exercises/practice between sessions (e.g., to process things that were triggering in the prior session, to engage in at-home application of skills learned, etc.). Participants in previous studies have emphasized that discussing IPV-related events is highly personal and understandably upsetting [50]; as such, it may be important to offer multiple sessions to allow for processing the difficult emotions that are often activated when discussing IPV and related situations. RISE’s multi-session format and client-centered strategies are aligned with trauma-informed counseling and other multi-session evidence-based trauma focused therapies (e.g., Prolonged Exposure [PE]) [64] that include between session practice to consolidate and further treatment gains. RISE was designed not to elicit trauma-focused details from survivors of IPV, but instead to provide a trauma-informed, non-judgmental intervention where women can discuss their IPV history in broad strokes while focusing on developing skills to prevent future IPV experiences, and enhancing empowerment and self-efficacy, both of which are primary factors that may be associated with long-term benefits in the lives of women [38]. 

### Limitations of the Study

Our findings must be interpreted in light of its limitations. First, the study’s modest sample size and relatively brief and limited follow-up assessments (10- and 14-weeks following enrollment) are limitations. The brief timeframe for the follow-up assessments makes it impossible to know if the usefulness and acceptability of the interventions persist over time. It is possible women may have different perceptions of the interventions’ strengths and weaknesses over time and that the 4 weeks between the two follow-up assessments was not long enough to capture such experiences. Future studies should include longer term follow-up assessments that include both qualitative and quantitative outcomes. Additionally, by design, there is variation in the uptake of the number of RISE sessions received and in the modules selected by participants. In the present study, the qualitative interviews were designed to assess participants’ experience with the RISE and advocacy-based ECAU interventions overall. The present study did not examine individual differences in how participants were impacted by the interventions and thus findings should not be interpreted to imply that all participants were affected the same by the intervention. Future studies of RISE should assess more specific feedback about the individual modules, ordering of the modules, and length of the RISE intervention. Although we found strong support among this sample for the identified themes, the findings are not necessarily universal, and the findings should not be assumed to apply to all women who experience IPV. Rather, these findings might highlight the importance of these themes as they relate to women Veterans and some themes may not be as transferrable to other clinical populations. Indeed, in addition to IPV, women Veterans tend to experience more trauma exposures over the lifespan and have additional mental and physical health needs as compared to their civilian counterparts [65,66]. Additionally, women Veterans who use the VHA often have mental and physical health conditions [67]. This is an important consideration since all participants were current VHA patients. Finally, the sample was limited to women, and future studies would benefit from including men and non-binary gender identities, who also experience IPV.

## 5. Conclusions

Understanding women’s experiences and preferences for psychosocial counseling interventions for IPV is critical to providing evidence-based and trauma-informed care for this prevalent issue. Our study focused on women Veterans. The current findings reinforce the notion that women want flexibility and a sense of empowerment surrounding the length and content of their IPV treatment. Additionally, it is important for clinicians to provide social and logistical support (tailored resources, concrete skills, and tools to apply in their daily lives), which helps women feel less alone in their IPV experiences. These are practical suggestions that can be implemented into clinical practice with women Veterans who experience IPV and are likely applicable to other populations of women experiencing IPV. 

## Figures and Tables

**Table 1:** Baseline Participant Characteristics by Condition (*N* = 58).

Sociodemographic Characteristics ^a^	RISE (*n* = 28)	ECAU (*n* = 30)	Statistic	*p*-Value
Age, *M* (*SD)*	38.0 (11.0)	40.5 (13)	*t* = 0.79	0.44
Race/Ethnicity			χ^2^ = 4.60	0.47
Black	7 (25.0)	5 (16.7)		
White/Caucasian	19 (67.9)	16 (53.3)		
Asian	1 (3.6)	2 (6.7)		
Native American	0 (0)	1 (3.3)		
Other Race	0 (0)	3 (10)		
Multiple Races	1 (3.6)	4 (13.3)		
White Non-Hispanic	27 (96.4)	25 (83.3)	χ^2^ = 0.21	0.64
Non-White/Hispanic	1 (3.6)	5 (16.7)		
Sexual Orientation			χ^2^ = 2.24	0.53
Heterosexual	18 (64.3)	24 (80)		
Lesbian/Gay	3 (10.7)	1 (3.3)		
Bisexual	5 (17.9)	4 (13.3)		
Pansexual	2 (7.1)	1 (3.3)		
Relationship Status			χ^2^ = 6.13	0.29
Married/Cohabitating	7 (25)	5 (16.7)		
LT/NM	7 (25)	5 (16.7)		
NM/NLT	3 (10.7)	1 (3.3)		
Single	7 (25.0)	8 (26.7)		
Separated	2 (7.1)	9 (30)		
Other	2 (7.1)	2 (6.7)		
Income			χ^2^ = 8.59	0.28
Less than $15,000	2 (7.1)	4 (13.3)		
$15,000–$24,999	2 (7.1)	4 (13.3)		
$25,000–34,999	3 (10.7)	3 (10)		
$35,000–$44,999	5 (17.9)	2 (6.7)		
$45,000–$54,999	3 (10.7)	6 (20)		
$55,000–$64,999	2 (7.1)	5 (16.7)		
$65,000–$74,999	3 (10.7)	0 (0)		
$75,000 or more	8 (28.6)	5 (16.7)		
Employment Status			χ^2^ = 28.15	0.001
Employed Full Time *	16 (57.1)	8 (27.6)		
Employed Part Time	5 (17.9)	4 (13.8)		
Student Full Time	4 (14.3)	7 (24.1)		
Student Part Time	2 (6.9)	1 (3.4)		
Unpaid Volunteer	3 (10.7)	7 (23.3)		
Retired or Other	5 (19.9)	4 (13.8)		
Education			χ^2^ = 3.48	0.63
Vocational/Tech College	3 (10.7)	5 (16.7)		
Some College/Associate	14 (50)	16 (53.3)		
Bachelor’s Degree	6 (21.4)	4 (13.3)		
Master’s/Doctoral Degree	5 (17.9)	5 (16.7)		
Military Branch			χ^2^ = 4.40	0.49
Army	15 (53.6)	16 (53.3)		
Navy	3 (10.7)	4 (13.3)		
Air Force	5 (17.9)	2 (6.7)		
Marine Corps	4 (14.3)	4 (13.3)		
Years of Military Service *M* (*SD)*	6.9 (5.6)	7.1 (8.9)	*t* = 0.09	0.93
IPV Experience			χ^2^ = 3.31	0.19
Past-Year Psychological IPV	28 (100)	30 (100)		
Past-Year Physical IPV	16 (57.1)	24 (80)		
Past-Year Sexual IPV	15 (53.6)	11 (36.7)		
Length of IPV in Relationship			χ^2^ = 3.00	0.81
Less than 6 months	6 (21.4)	3 (10)		
Between 6 months and 1 year	6 (21.4)	5 (16.7)		
Between 1 and 3 years	6 (21.4)	6 (20.0)		
Between 3 and 5 years	4 (14.3)	5 (16.7)		
Between 5 and 7 years	2 (7.1)	2 (6.7)		
Between 7 and 9 years	0 (0)	1 (3.3)		
10+ years	4 (14.3)	7 (23.3)		

Note. * In the Enhanced Care as Usual (ECAU) condition, the participant who missed the 10–week assessment was not the same participant who missed the 14-week assessment. Thus all 30 ECAU participants had interview data analyzed in the current study. ^a^ All values are n (%) unless otherwise specified. Percentages may not equate to 100% because of rounding and/or missing data for participant characteristics. Military characteristics are for Veteran participants (*n* = 55; condi-tions did not differ in proportion of non-Veteran participants; *p* = 0.16). Abbreviations: RISE = Recovering from IPV through Strengths and Empowerment, ECAU = enhanced care as usual, LT/NM = living together/not married, NM/NLT = not married/not living together.

## Data Availability

The datasets generated during and/or analyzed during the current study are not publicly available due to Human Studies protections placed upon them by the Boston VA Healthcare System Institutional Review Boards. Data are available from the authors upon reasonable request, which would also involve obtaining permission from the VA Boston Healthcare System Institutional Review Board.

## Trial Registration

ClinicalTrials.gov Identifier: NCT03261700.
